# On *Pulchritia* new genus, with a reappraisal of the genera of Trichotriidae (Rotifera, Monogononta)

**DOI:** 10.3897/zookeys.342.5948

**Published:** 2013-10-14

**Authors:** Yongting Luo, Hendrik Segers

**Affiliations:** 1 Department of Biology, Shanghai Normal University, Guilin Road 100, Shanghai, P.R.China; 2 Royal Belgian Institute of Natural Sciences, Vautierstraat 29, B 1000 Brussels, Belgium

**Keywords:** Africa-South America vicariance, biogeography, *Macrochaetus*, taxonomy

## Abstract

During the study of rotifers collected in Eastern DR Congo, we rediscovered specimens that correspond to *Monostyla dorsicornuta* Van Oye, 1926. This species, which we redescribe, had not been seen since it’s summary description, and lacked type material. Our analysis reveals that the animal belongs to Trichotriidae rather than to *Lecane* (presently considered to include *Monostyla*) or Lecanidae, but is nevertheless characterised by a foot structure that is remarkably convergent to that of Lecanidae, and different from all other genera of Trichotriidae. We conclude that the species and the closely related South American *Macrochaetus kostei* (José de Paggi, Branco & Kozlowsky-Suzuki, 2000) belong to a new genus of Trichotriidae; the two offer a rare example of African-South American vicariance in rotifers.We further provide emended diagnoses of the remaining genera of Trichotriidae, to conform these to the new information and to address some inconsistencies in these.

## Introduction

On the occasion of the 2010 International Year of Biodiversity and the 50th anniversary of the independence of the Republic of Congo, an international expedition explored swamps, rivers and other water bodies along a ~1750km stretch of Congo River Northwest of Kisangani (Congo Biodiversity Initiative: Expedition 2010). The expedition offers a unique opportunity to study the taxonomy and biogeography of organisms from a relatively inaccessible region in Central Africa. To date, very little information on the micrometazoa of the region, especially Rotifera, exists. There is fragmentary information dating from the first half of the 20^th^ Century (reviewed in Gillard 1957, De Ridder 1986), but very little additional data are available.The dearth of information on the old and climatically relatively stable Congo Basin probably lies at the origin of Dumont’s (1983) observation, that the African continent is outstanding for its apparently poor and uncharacteristic rotifer fauna. This “African anomaly”, as Dumont (1983) named it, has already been partly refuted by studies on floodplain lakes from the River Niger in Nigeria (Segers 1993, Segers et al. 1993) and by results from isolated studies describing new endemic species from different localities in Cameroon (Segers and Mertens 1997) and Kenya (Segers et al. 1994, see De Ridder 1991, 1994 for an overview of recent African records of rotifers), but studies on the Congo River Basin remained scarce: only De Smet (1988, 1989) provides detailed accounts on the rotifer fauna of freshwater habitats in the Bas Zaïre.

The samples collected during the 2010 International Congo River expedition contained an abundance of rotifer material. It also contained numerous specimens of what we believe to be an enigmatic species of which only a brief description by Van Oye (1926) exists. In the present paper we provide a redescription of the taxon, and further considerations on its phylogenetic and biogeographic significance.

## Material and methods

As mentioned before, the material of this study consists of samples collected during the 2010 International Congo River Expedition. Specimens of the target taxon were found in three qualitative, 4%-formaldehyde-preserved samples only, all from running water in rivers: sample KM-028 is from Lulu River near Basoko, KM-048 and KM-049 are from Lohulu River near Bomane, all DR Congo. The samples were collected by Papy Mongindo, Ernest Tambwe and Koen Martens using a either a 30- or a 50 µm mesh-width plankton net that was hauled through surface water (maximum 1 m depth) and the littoral.

Individual rotifer specimens were separated under a WILD M10 dissection microscope and examined and measured on an Olympus BX51compound microscope at high magnification using a micrometre eyepiece. Drawings were made using a camera lucida. Photographs were taken by a camera (Olympus C-5060) connected to the microscope. Stacks of photographs were combined used COMBINEZP (Hadley 2010). Materials are deposited in the Royal Belgian Institute of Natural Sciences, Brussels, Belgium (RBINS), and the Centre de Surveillance de la Biodiversité, University of Kisangani, Kisangani, DR Congo (CSB-UK).

## Results and discussion

**Figure 1.**
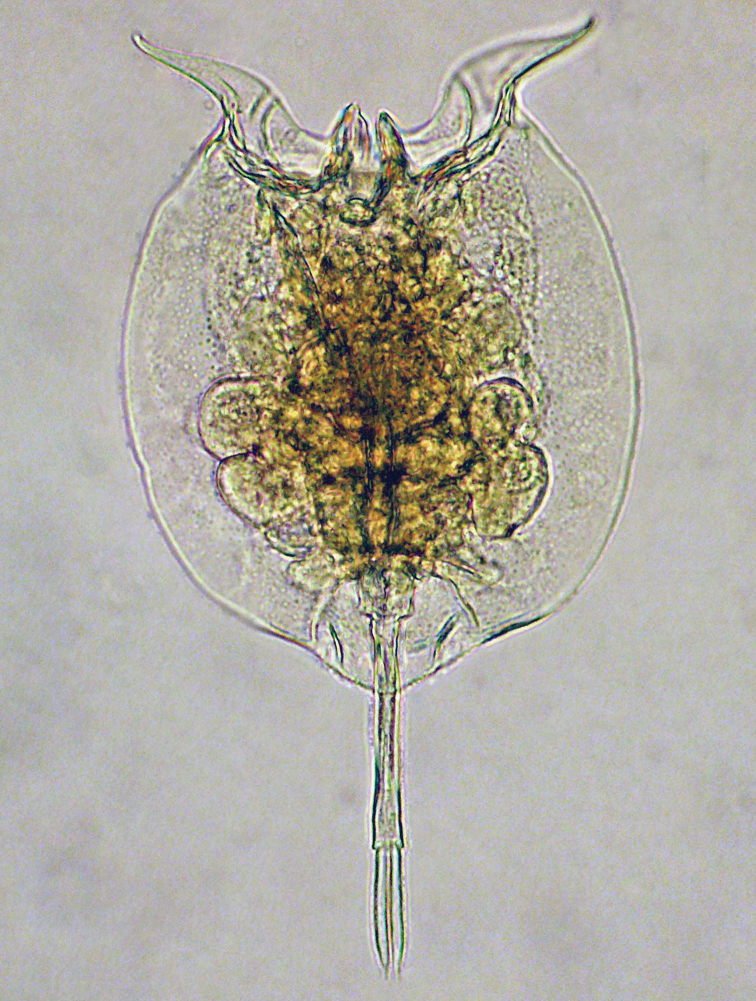
*Pulchritia dorsicornuta* gen. n., comb. n., compound photomicrograph.

**Figures 2.**
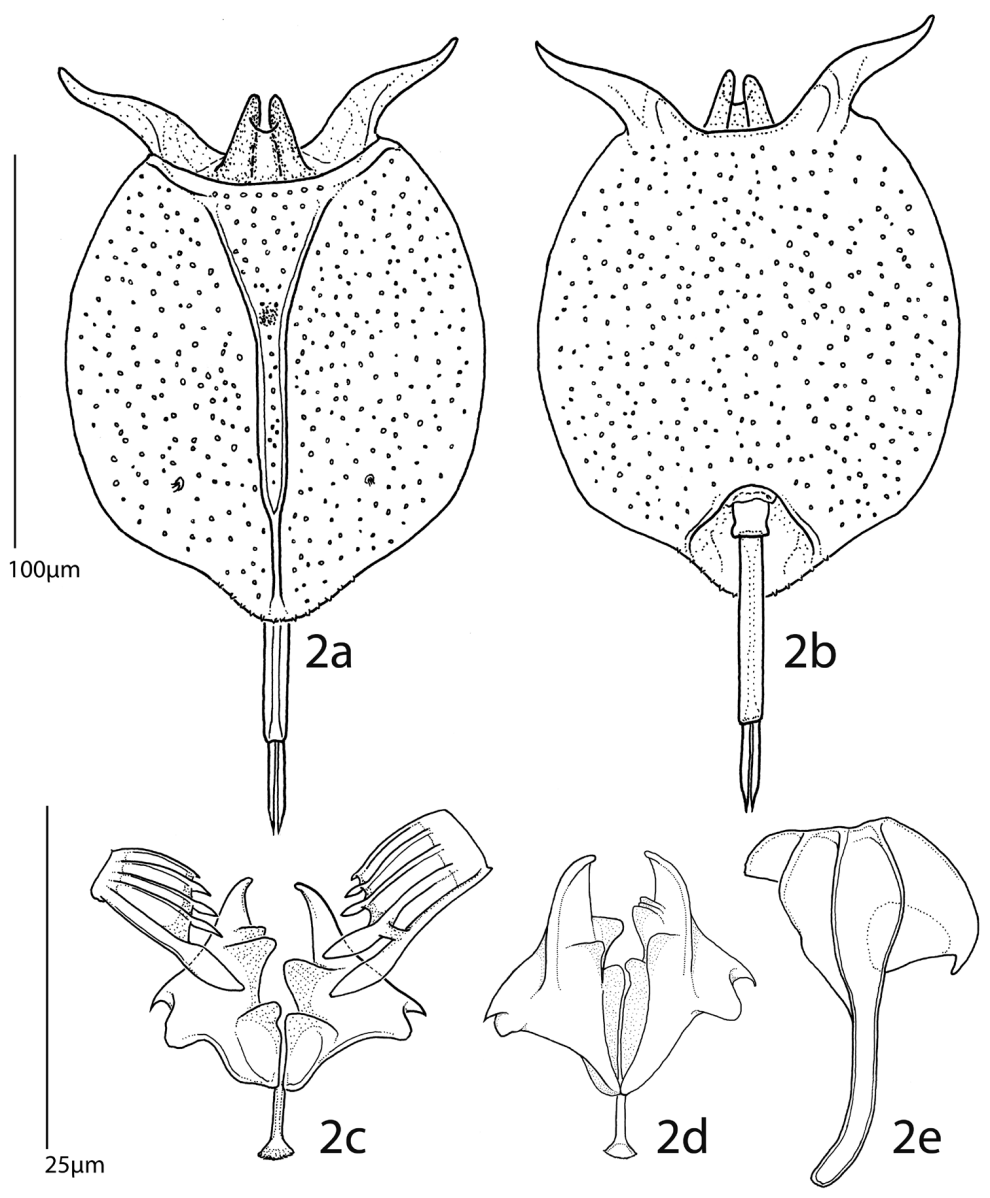
*Pulchritia dorsicornuta* gen. n., comb. n., **a** habitus, dorsal **b** habitus, ventral **c–e** trophi **c** unci and incus, frontal **d** incus, caudal **e** left manubrium, external. Scale bars: **a–b**= 100µm, **c–e**= 25µm.

We found numerous specimens of our target species (Figs 1–2) which we identify, with some hesitation, as the species described as *Monostyla dorsicornuta* Van Oye, 1926, from the Ruki River near Eala, Congo. This description lacks detail and was considered to be based on some unrecognisable, poorly contracted rotifer by the authors of the candidate Rotifera part of the List of Available Names in Zoology (Jersabek et al. 2012, Segers et al. 2012). When compared to the present material, it indeed probably concerns a poorly contracted specimen. In a footnote to the rather brief original description of this animal, P. de Beauchamp noted that “Il est regrettable que des préparations n’aient pas été conservées…“, indicating that no types were deposited. Nevertheless, the general round lorica shape with large antero-lateral spines, shape of retracted head, and foot and toe shape of *Monostyla dorsicornuta* correspond to our material, albeit that the present specimens are slightly smaller in size than reported for *Monostyla dorsicornuta* by Van Oye (1926). In view of the poor original description and significance of the species we suggest to stabilize the taxonomic status of this nominal taxon name by designating a neotype for *Monostyla dorsicornuta* (see further).

By its trophi and lorica structure the species does not belong to Lecanidae as defined by Segers (1995). Following the key by Koste (1978), the animal keys out to Trichotriidae. Assigning the species to one of the three recognized genera appeared problematic due to inconsistencies in these definitions, and the peculiar morphology of the specimens treated here. This prompted us to the following reassessment of the diagnosis of Trichotriidae and its genera.

### Family Trichotriidae

Harring, 1913

http://species-id.net/wiki/Trichotriidae

#### Diagnosis.

Trophi unspecialized, malleate; head, trunk and foot largely loricate, but head retractable. No discernible separate lorica plates or sulci on the trunk, but lorica stiffness not homogeneous. Lorica granulated and/or facetted. Distal part of trunk (anal segment) illoricate, separated from trunk proper. Foot with two pseudosegments and a pair of terminal toes.

#### Discussion.

The diagnostic autapomorphic feature for the family is the stiffening of the tegument of the head region, especially of the neck and lateral parts of the head, which in contracted specimens folds into a characteristic, more or less symmetrical shape protruding from the head aperture. The feature distinguishes family members from Brachionidae, Epiphanidae, Euchlanidae, and Mytilinidae who have an illoricate head; Lepadellidae has a characteristic sclerotized head shield overlaying the corona but the rest of the head is illoricate (*Colurella*, *Lepadella*), and not retractile (*Squatinella*). In contrast, Sørensen and Giribet’s (2006) detailed phylogenetic analysis could not confirm monophyly of Trichotriidae, neither on molecular nor morphological data.

The classic diagnoses of Trichotriidae genera are problematic. They refer to features that are not present in all species of the genus (e.g., the purported synapomorphic dorsal spines in *Macrochaetus*), or features which appear to have been misinterpreted. This holds in particular for the structure of the foot which, in its basic form, consists of two foot pseudosegments bearing two toes, and is inserted on an illoricate terminal part of the trunk, termed the anal segment. This anal segment is a part of the trunk proper as it lies anterior to the (dorsal) anal opening. Its tegument is always relatively weakly sclerotized, which enables mobility of the rigid foot relative to the rigid trunk lorica, but which may also make it difficult to distinguish it from the trunk and/or from the two distal pseudosegments of the foot in contracted specimens. The structure is occasionally mistaken for a part of the foot and is then referred to as first of three foot pseudosegments. Note that in Koste and Shiel (1989) both terms (anal segment and first foot segment) appear to have been used for the same structure, and that the position of the anus as indicated in their fig. 16:1 is incorrect.

In view of these inconsistencies, and awaiting a full review, preferably integrating both molecular and morphological data of genera in this and the related Euchlanidae and Mytilinidae, we tentatively propose emended diagnoses of the trichotriid genera, and propose a new genus to accommodate *Monostyla dorsicornuta* Van Oye, 1926 and *Macrochaetus kostei* José de Paggi, Branco & Kozlowsky-Suzuki, 2000.

### Genus Pulchritia gen. n.

http://zoobank.org/8D9FFA3E-E5D5-4D71-8D3B-479F4700FF26

http://species-id.net/wiki/Pulchritia

#### Type species.

*Monostyla dorsicornuta* Van Oye, 1926.

#### Diagnosis.

Body, including head and foot, loricate; head retractile, foot non-retractile, consisting of a short basal, squarish and an elongate, cylindrical foot pseudosegment terminating in two equal toes. Anal segment strongly reduced. Trunk lorica ventrally relatively flat, dorsally with a Y-shaped keel, pustulated, rounded elliptical.

#### Etymology.

The name *Pulchritia* is derived from the Latin adjective *pulcher*, meaning “pretty, beautiful, handsome”. It refers to the beauty of its type species, *Pulchritia dorsicornuta* comb. n.

#### Discussion.

We recognize this genus as containing two species, *Pulchritia dorsicornuta* comb. n. and *Pulchritia kostei* (José de Paggi, Branco & Kozlowsky-Suzuki, 2000), comb. n.

The two share a number of features that clearly sets them apart from other Trichotriidae. Their rounded, dorso-ventrally flattened trunk shape reminds one only of *Macrochaetus*, while the anal segment being reduced is as in certain *Trichotria* (e.g., *Trichotria buchneri* Koste, Shiel & Tan, 1988, *Trichotria brevidactyla* Harring, 1913 (= *Trichotria curta* (Skorikov, 1914))). The peculiar keel formation of the dorsal lorica is somewhat similar to *Trichotria buchneri* only. The unique foot structure of the two species, however, can be considered synapomorphic and is superficially and probably functionally similar to the foot consisting of a single short foot pseudosegment and elongated, fused toes bearing terminal (pseudo)claws of some *Lecane* species.

### Redescription of Pulchritia dorsicornuta

(Van Oye, 1926) comb. n.

http://species-id.net/wiki/Pulchritia_dorsicornuta

#### Material examined.

Type material: Neotype (labelled: “*Pulchritia dorsicornuta* (Van Oye, 1926) Neotype. Lohulu River near Bomane, DR Congo, 24 May 2010 (KM-048)”) in Royal Belgian Institute of Natural Sciences, Brussels Belgium (IG32450, RIR 212).

Other material: Abundant specimens of the species were found in two localities: Lulu River near Basoko (sample KM-028: 1.2958°N, 23.6497°E (DD, GPS waypoint Mac 079), altitude. ca. 350 m asl., water temp. 25.8° C, conductivity 16.5 µS/cm), and Lohulu River near Bomane (samples KM-048, KM-049: 1.2486°N, 23.7280°E (DD, GPS waypoint Mac 089), altitude. ca. 410 m asl., water temp. 24.3° C, conductivity 30.4 µS/cm, oxygen 0.45 mg/l), both in Orientale province, DR Congo. All samples are from running water. One permanent trophi preparation, and nine permanent slides containing one, three slides containing two, and three slides containing three specimens. Deposited in RBINS and in the CSB-UK.

#### Diagnosis.

*Pulchritia dorsicornuta* comb. n. is unmistakable by the large, S-shaped antero-lateral projections of its ventral lorica. These are completely absent in its closest relative *Pulchritia kostei* comb. n.

#### Description.

Female (Figs 1, 2a–b; male unknown): Body: Head largely retracted in trunk lorica, with two lateral stiffened elements protruding from the head aperture. A pigmented spot (eye?) present. Trunk loricate, elliptic in outline, longer than wide, dorso-ventrally compressed. Ventral and dorsal plates fused laterally and caudally, leaving a broad head aperture and a smaller foot aperture. Dorsal plate medially with two semi-longitudinal ridges forming a Y-shaped double dorsal keel, fused to a single dorsal keel terminally. Posterior of dorsal lorica with a weakly protruding rounded margin bearing two pairs of short ridges over the foot aperture. Openings of the lateral antennae in posterior third of body, about halfway between dorsal keel and lateral margin of lorica. Dorsal head aperture margin concave. Ventral plate flat, with two protruding, weakly S-shaped and diverging spines antero-laterally, these separated by a shallow U-shaped sinus. Posterior of ventral plate with a well-defined foot aperture, with rounded anterior and diverging lateral margins. Anal segment indistinct, poorly developed (also in poorly contracted specimens). Foot subterminally, consisting of a short, bilaterally constricted first and an elongate, parallel-sided second foot pseudosegment. Two long, equal toes, these mostly parallel-sided, terminating in a sharp tip.

Trophi (Figs 2c–e) malleate, almost symmetrical. Fulcrum short, with a small basal plate; rami relatively flat, triangular, with rounded postero-lateral corners and short, curved alulae, inner margins with asymmetrical, protruding teeth-shaped structures. Left uncus with two large frontal and three minor dorsal webbed teeth, right with a single large frontal and four minor teeth, all minor teeth gradually reduced in size from frontal to dorsal. Manubria symmetrical, with elongate and weakly procurved shaft. Head broad, with clear ventral, median and dorsal chambers, anterior chamber with an additional rounded triangular apophysis, dorsal chamber with a recurved hook.

#### Measurements

(in µm. N=12; range, mean).Total length (incl. foot): 180–205, 192; lorica width 92–122, 106; antero-lateral spine length 20–32, 27; head aperture width 37–58, 47; foot aperture width 29–40, 33; length 23–34, 28; first foot pseudosegment length 9–14, 11; second foot pseudosegment length 46–54, 48; toe length 26–32, 29.

#### Distribution.

*Pulchritia dorsicornuta* comb. n. is only known from the two localities cited above, and from Ruki River near Eala (Van Oye 1926), near Mbandaka, Equator province, DR Congo. Its close relative *Pulchritia kostei* co*m* b. n. is known only from a coastal lagoon, State of Rio de Janeiro, Brazil. We hypothesize that the two represent a vicariant species pair. This is remarkable as there are few examples of such vicariant sister-taxa, possibly originating from allopatric speciation, in rotifers, and patters are blurred by their purportedly superb dispersal potential (Segers 2008, Segers and De Smet 2008). Some have been identified before in the genus *Lecane* (see Segers 1996), but the most notorious example of such a vicariant species-pair is *Kellicottia longispina* (Kellicott) and *Kellicottia bostoniensis* (Rousselet), in which the former is hypothesized to be of Palaearctic, the latter of Nearctic origin (Pejler 1977).

#### Comments.

The main feature distinguishing *Pulchritia dorsicornuta* comb. n. and *Pulchritia kostei* comb. n. is the presence of well-developed antero-lateral spines in the former. As we observed only negligible variability of the antero-lateral spines of *Pulchritia dorsicornuta* comb. n., and as there are no indications at all of such spines in *Pulchritia kostei* comb. n., we can neither exclude nor confirm the possibility that this feature results from phenotypic plasticity and as such would not be taxonomically relevant. Examples of such environmentally induced spine development are common in rotifers, including Trichotriidae (Gilbert 2011a, 2011b, Koste 1978, Luo et al. 2012, Wallace et al. 2006). We prefer to remain cautious and treat the two as separate taxa, pending proof to the contrary.

### Genus Macrochaetus

Perty, 1850

http://species-id.net/wiki/Macrochaetus

#### Type species.

*Macrochaetus subquadratus* Perty, 1850.

#### Emended diagnosis.

Body, including head and foot, loricate; head retractable, foot not retractable, inserted on a large, relatively soft and broad anal segment covering an equally soft and relatively broad first foot pseudosegment, and a stiff, cylindrical terminal foot pseudosegment. Trunk lorica dorso-ventrally compressed, relatively wide, pustulated, circular or with angular corners in the anterior third, head and neck lorica plates with spinulets.

#### Discussion.

Most species of *Macrochaetus* are readily identified as belonging to this genus by the presence of long, conspicuous dorsal spines. However, three species of *Macrochaetus* (*Macrochaetus aspinus* Segers & Sarma, 1993, *Macrochaetus danneelae* Koste & Shiel, 1983, and *Macrochaetus paggiensae* Koste, 2000) lack these dorsal spines and their presence can therefore not be confirmed as generally diagnostic for the genus. On the other hand, small lorica spinulets are present dorsally, ventrally and marginally on the trunk lorica, and on the lorica of the head and neck regions. In particular the spinulets on the head and neck lorica appear to be synapomorphic for the genus. The foot consist of a large, relatively soft anal segment covering a relatively poorly sclerotized first foot pseudosegment and a terminal cylindrical foot pseudosegment bearing two separate toes. There are 14 species in this genus, several of which are endemic to South America (Segers 2007, Segers and De Smet 2008).

### Genus Trichotria

Bory de St Vincent, 1827

http://species-id.net/wiki/Trichotria

#### Type species.

*Trichotria pocillum* (Müller, 1776).

#### Emended diagnosis.

Body, including head and foot, loricate; head retractable, foot only partly retractable. Trunk lorica hexagonal in cross section, facetted, granulated, longer than wide, with parallel lateral margins in anterior part of the trunk. Head aperture nearly as wide as the trunk.

#### Discussion.

In comparison with *Wolga*, the anal segment is clearly discernible in almost all species but it is relatively weakly sclerotized; the two foot pseudosegments are cylindrical and strongly sclerotized. Retraction of the foot is not possible in those species in which the foot is situated terminally. Spines on the first foot pseudosegment and on the trunk lorica are present in most, but not all species (e.g., *Trichotria pseudocurta* Koste, Shiel & Tan, 1988). There are seven species in the genus (Segers 2007), most are cosmopolitan, one (*Trichotria brevidactyla*) is Holarctic, two are Australian. Regarding the latter, however, the attribution of *Trichotria buchneri* to *Trichotria* was considered uncertain by Koste and Shiel (1989) in view of this species’ peculiar triangular cross section, and foot consisting of two cylindrical pseudosegments only (apparently without, a reduced, or completely retracted anal segment in the preserved material examined?). A re-examination of the species is in order.

### Genus Wolga

Skorikov, 1903

http://species-id.net/wiki/Wolga

#### Type species.

*Wolga spinifera* (Western, 1894).

#### Emended diagnosis.

Tegument of head, and anterior part of trunk loricate, both head and foot entirely retractable. Anal segment relatively large, annulated; foot pseudosegments short, only the distal one sclerotized. Trunk lorica box-shaped, dorso-ventrally compressed, longer than wide, with relatively flat ventral and dorsal parts; facetted. No marginal spines or spinulets.

#### Discussion.

The published generic diagnosis refers to absence of an anal segment (Koste 1978; Koste and Shiel 1989). This does not appear to be correct; the illustration of a non-contracted animal by Western (1894, reproduced by Koste (1978) and Koste and Shiel (1989)) depicts a foot consisting of a first element having numerous transverse folds indicating its high mobility, and two additional, relatively short pseudosegments; all but the terminal part may be indistinct in preserved specimens. We interpret the first element as being the anal segment. The foot is situated subterminally and can be retracted entirely into the lorica by which the anal segment becomes indiscernible; there are two separate toes. It is unlikely that the presence of short spines over the lateral antennae is diagnostic at the genus level.

Western (1894) notes that the lorica of the species would consist of plates connected with a membranous lateral invagination. While the lateral parts of the trunk lorica may be concave, they do not appear distinctly less sclerotized as in, e.g., many species of *Lecane*.

## Supplementary Material

XML Treatment for Trichotriidae

XML Treatment for Pulchritia

XML Treatment for Pulchritia dorsicornuta

XML Treatment for Macrochaetus

XML Treatment for Trichotria

XML Treatment for Wolga
